# Social Media Use for Health Communication by the CDC in Mainland China: National Survey Study 2009-2020

**DOI:** 10.2196/19470

**Published:** 2020-12-02

**Authors:** Runxi Zeng, Menghan Li

**Affiliations:** 1 Center for Communication and Social Development School of Journalism and Communication Chongqing University Chongqing China

**Keywords:** social media, public health agencies, Center for Disease Control and Prevention, China, government Weibo, COVID-19

## Abstract

**Background:**

In recent years, public health incidents that pose a serious threat to public life have occurred frequently in China. The use of social media by public health authorities has helped to reduce these threats by increasing effective risk communication between the government and the public.

**Objective:**

The aim of this study is to reveal how China’s Center for Disease Control and Prevention (CDC) uses social media to improve three aspects of health communication between the government and the public: adoption, operation, and interaction.

**Methods:**

To analyze the 134 CDC government Weibo accounts at the provincial- and prefecture-level administration regions in mainland China, we collected their account data and extracted 1215 Weibo tweets. We also supplemented the data to reveal the overall performance of the CDC’s government Weibo use during the COVID-19 crisis.

**Results:**

The registration rate of the CDC’s government Weibo accounts increased year by year, and the local authorities registered Weibo accounts before the central government authorities. In total, 29.8% (n=134) of the 450 CDC facilities have registered an account. Among the 134 CDC facilities that have registered Weibo accounts, the registration rate in the eastern region (n=68, 50.7%) was higher than those in the central region (n=30, 22.4%) and the western region (n=36, 26.9%). Nearly 90.0% of these Weibo accounts had official certification, but there were dropouts in the specific operating process. One-third of the accounts have not been updated for more than 1 year, and the number of Weibo followers was polarized, with a maximum and minimum difference of 1 million. The response rate to users’ comments was less than 1%. Emergency information, multimedia content, and original content were more helpful in promoting communication between the government and the public. Such interaction was partially improved during the COVID-19 pandemic. The CDC updated the daily epidemic situation and provided popular science information for epidemic prevention and control for the public in a timely manner.

**Conclusions:**

China’s CDC is using more social media to popularize daily health information and has taken the first step to improve communication between the government and the public. However, equal dialogue, two-way interactions, and effective communication with the public still need improvement.

## Introduction

One of the keys to dealing with public health emergencies is timely and effective risk communication. Through a dialogue between the government and the public, we can minimize the information asymmetry between the government and the public, and help the public to take preventive measures quickly [1]. With the development of information and communication technology, social media, with the characteristics of participation, openness, dialogue, and other tools, has brought unprecedented opportunities for improving communication between the government and the public [2,3]. Thus, social media can improve the ability of government management to strengthen communication between the government and the public, and to realize an open government [4]. For example, previous studies have shown that the use of social media by the government can promote cross-sectoral information sharing [5], increase government transparency [6], promote public political participation [7], and help the public to respond to disasters [8].

Public health authorities are also increasingly using social media for information disclosure and risk communication. Previous studies have analyzed the main factors of public health authorities’ willingness to adopt social media, such as organizational size and geographical location [9,10]. Studies have also shown that traditional media is used by public health authorities to respond to general health problems, while social media is used to respond to public health emergencies [11], and this role of responding to public health emergencies has been confirmed by other studies [12-14]. For example, analyzing the number of tweets and the degree of public participation can effectively predict the actual dynamics of an epidemic [15]. In addition, existing research also discusses the role of social media use by public health authorities in advancing health reform and building an open government [16,17]. In general, existing studies have explored the influencing factors, preferences, and significance of social media use, but they have paid less attention to how public health authorities use social media to communicate with the public. In particular, these studies have rarely investigated and evaluated the effects of communication [18].

Moreover, in the Western context, China is regarded as an authoritarian country that differs from the Western-style democratic system. Sudden public health incidents occur frequently in China and have an impact on China’s economic development and social stability. Previous studies have attributed these effects to China’s administrative system. These studies have noted that China’s emergency decision making is often guided by a top-down command and control system, and that information transmission follows a layer-by-layer linear model. As a result, the public feedback channel is not smooth, and the interaction between the government and the public is limited [19,20]. For example, during the severe acute respiratory syndrome incident, there were delays in decision making caused by poor communication, which led to the spread of public panic and threatened public health and safety [19]. This situation has also occurred in the fight against COVID-19. Therefore, identifying ways to improve communication between the government and the public, and promoting timely and effective risk communication is key for China to deal with sudden public health incidents. However, there are few studies that have examined the current situation and the interaction of social media use by Chinese public health authorities from an empirical perspective.

The purpose of this study is to examine how Chinese public health authorities use social media to improve communication between the government and the public. This study analyzes the government affairs Weibo information of the Center for Disease Control and Prevention (CDC) at the provincial and prefectural levels in mainland China; describes the current situation of the adoption, operation, and communication of government Weibo accounts; discusses whether the use of social media by Chinese public health authorities has improved health communication between the government and the public; and discusses what factors help to promote communication between the government and the public. Specifically, this study aims to answer the following questions:

1. Is it common for the CDC at the provincial and prefectural levels in mainland China to use government Weibo accounts?
2. What is the current status of government Weibo use by the CDC at the provincial and prefectural levels in mainland China?
3. How does the CDC at the provincial and prefectural levels in mainland China communicate with the public on government Weibo accounts?

## Methods

### Study Sample

The CDC is a state-funded bureau under the leadership of the National Health Commission of China that specializes in disease control and prevention, and public health. According to the official website, its mission is to create a safe and healthy environment, maintain social stability, ensure national security and promote the health of people through the prevention and control of diseases, injuries, and disabilities. There are corresponding institutions from the central government to the local government. Sina Weibo is one of the most popular social media sites in China and is similar to Twitter. As of December 2019, there were 139,000 government agencies with registered Weibo accounts on the platform, according to the China Internet Network Information Center 45th Statistical Report on the Development of the Internet in China [21]. Because Weibo is highly influential, government agencies set up government Weibo accounts on the Sina Weibo platform.

A total of 134 sample accounts were collected. The sample collection process was as follows. First, through the “find-search-user” function on the Sina Weibo client, we conducted searches with “xx province + CDC” (“xx省+疾病预防控制中心”), “xx City + CDC” (“xx市+疾病预防控制中心”), “Centers for Disease Control and Prevention” (“疾控中心”), and “disease control and prevention” (“疾控”) as the keywords for the search. For example, “Jiangsu Centers for Disease Control and Prevention” (“江苏省疾病预防控制中心”), “Nanjing Centers for Disease Control and Prevention” (“南京市疾病预防控制中心”), and “Chongqing Disease Control and Prevention” (“重庆疾控”) are Weibo accounts that meet the requirements. Second, we visited the CDC’s official website and used the Weibo account announced by the CDC on its official website as a sample. The samples were collected from March 21, 2019, to March 30, 2019. To understand the use of the CDC’s government Weibo accounts during COVID-19, this study also observed the data for these 134 sample accounts from January 1 to June 30, 2020. To ensure that there were no omissions, the collection process was jointly undertaken by a teacher and two trained master’s degree students, and the collection results of the three were found to be completely consistent.

### Data Collection

**Figure 1:**
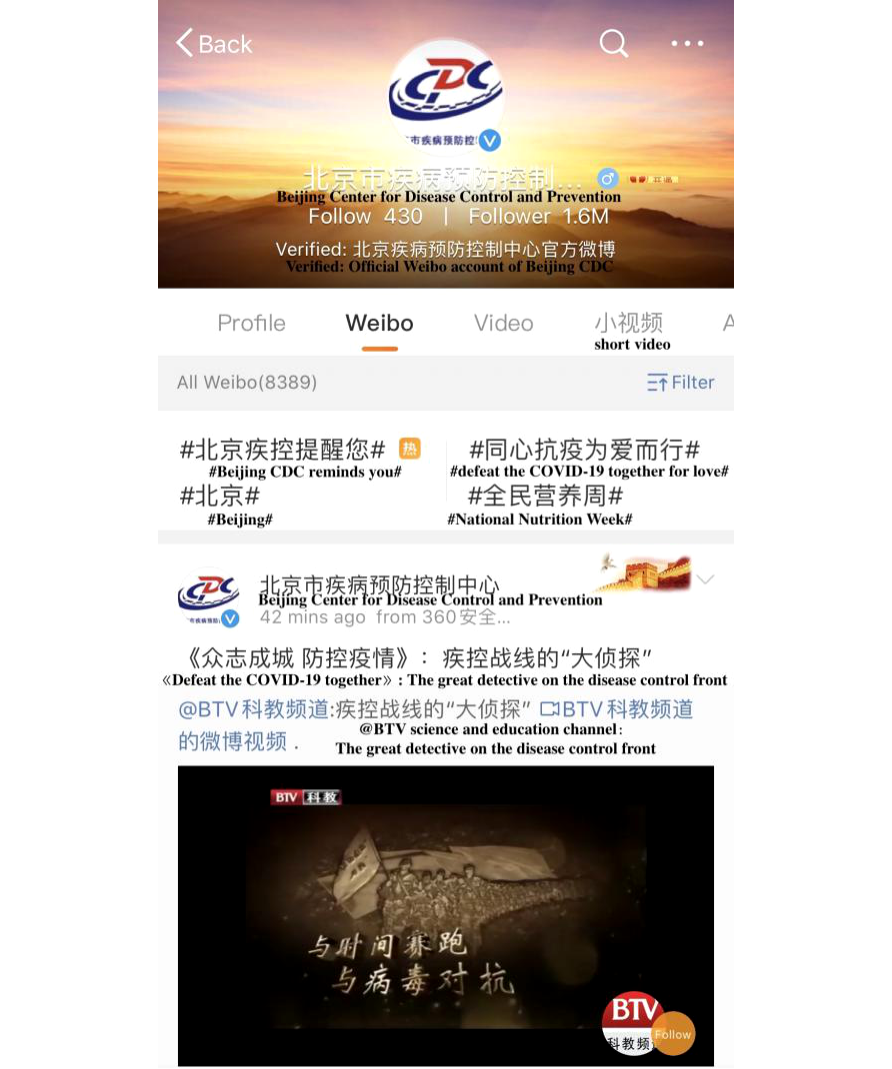
Sina Weibo webpage of a Center for Disease Control and Prevention facility in China.

Data collection for this study was mainly done using the Sina Weibo webpage. Figure 1 shows the Sina Weibo webpage of a CDC facility in China. We collected the latest 10 Weibo tweets posted by these 134 CDC government Weibo accounts before midnight on March 30, 2019. Considering that some Weibo accounts had posted less than 10 tweets or no tweets since their registration, we could not collect all 1340 tweets. Therefore, this study collected a total of 1215 Weibo tweets. The data collected mainly included topic type, content form, degree of originality, reply rate of comments, and reply time of comments. In addition, this study collected supplemental relevant data on the overall performances of the CDC’s government Weibo accounts in the pandemic from January 1 to June 30, 2020.

In this study, the adoption and operation of the CDC’s government Weibo accounts were also included in the survey. In the survey on the adoption of government Weibo accounts, the account registration time was collected. In the survey on the operation of government Weibo accounts, the data collected included the following: whether they received official certification from Sina Weibo, the update time for the most recent Weibo post, and the number of followers.

### Data Analysis

As China’s regional economy has shown uneven development in the eastern, central, and western regions [22], which may have an impact on the development of the CDC’s government Weibo accounts, this study is based on the division of the eastern, central, and western regions of mainland China by the National Bureau of Statistics. The CDC was divided into the “Eastern CDC,” “Central CDC,” and “Western CDC” for observation. In terms of data coding, first, whether the Weibo account passed the official certification of Sina Weibo was coded as passed or failed. Second, the year of registration of the CDC’s government Weibo accounts was coded as follows: 2009-2011, 2012-2014, 2015-2017, or 2018-2019. Third, the update time of the most recent Weibo post was coded as follows: within 30 days, 31-90 days, 91-365 days, more than 365 days, or no content. In the analysis of the government Weibo accounts’ interactions, this study used the content analysis method to code and analyze Weibo materials, as shown in Textbox 1. Before the formal coding, we analyzed the reliability of the three coders. The overall reliability was 0.97, and the lowest item reliability was 0.93, which met the reliability requirements of content analysis.

**Dimensions and indicators of 1215 Center for Disease Control and Prevention government Weibo tweets.**

**Topic type**

- Disease control information
- Emergency information
- Popularization of health knowledge
- Popularization of disease knowledge
- Radiation hygiene/school hygiene
- Government affairs trends
- Policy interpretation
- Weibo help/citizen consultation
- Other

**Content form**

- Only original text
- Posts with “pictures/videos/hyperlinks”

**Degree of originality**

- Original posts
- Retweeted posts

**Reply rate of comments**

- Reply only once
- Interactive reply

**Reply time of comments**

- Between 0 hours and 1 hour
- Between 1 hour and 8 hours
- Between 8 and 12 hours
- More than 12 hours

### Availability of Data and Materials

All the data are publicly available on the internet via the search strategy indicated in the Study Sample section. The original data are in Chinese and can be provided upon request.

### Ethics Approval and Consent to Participate

The study was reviewed and approved by the Academic Committee of the School of Journalism and Communication at Chongqing University, which acts as an ethics committee. According to the committee’s review report, the sample of this study are nonparticipants. Therefore, this study does not violate research ethics.

## Results

### Popularization of the CDC’s Government Weibo Accounts

#### Geographical Distribution of the CDC’s Government Weibo Adoption

**Figure 2:**
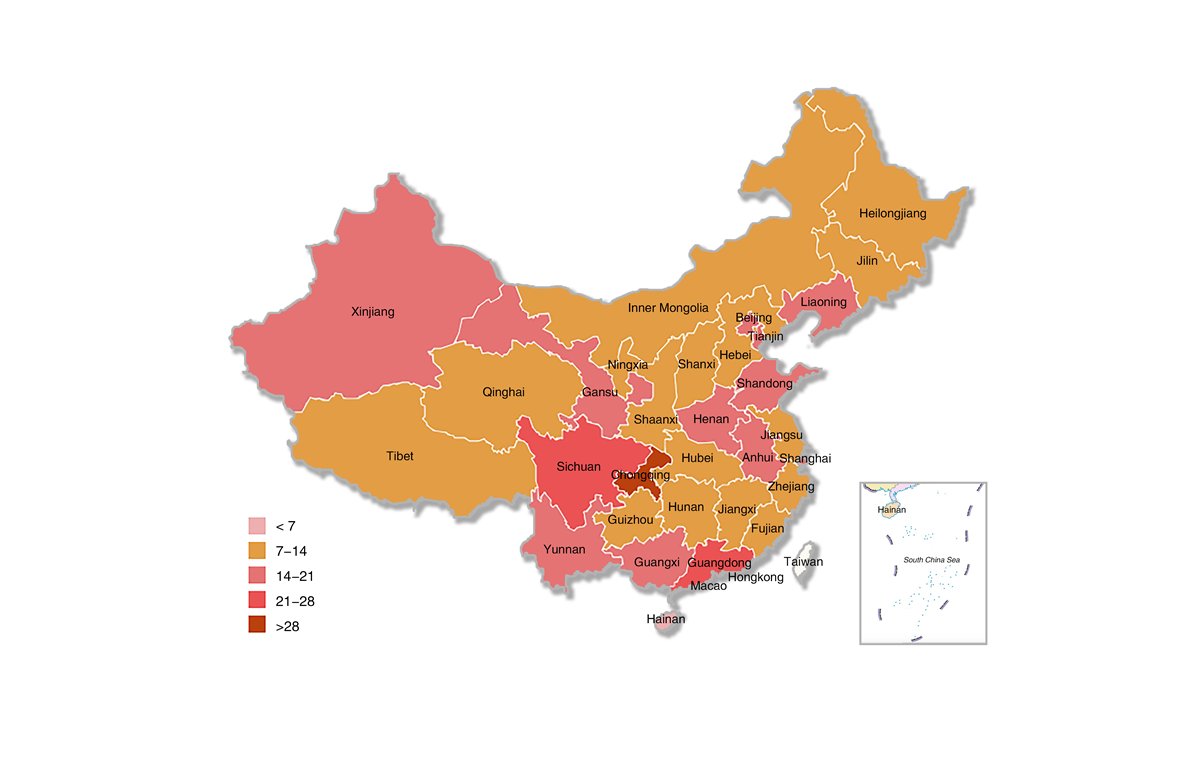
Distribution of the Center for Disease Control and Prevention facilities in mainland China.

There are a total of 450 CDC facilities in 31 provincial- and prefecture-level administrative regions in mainland China, as shown in Figure 2. In total, 31 provincial-level CDC government Weibo accounts should be registered, but only 8 have been registered, with a registration rate of 25.8%. Additionally, 419 prefectural-level CDC government Weibo accounts should be registered but only 126 have been registered, with a registration rate of 30.1%.

There are regional differences in the adoption of the CDC’s government Weibo accounts, and the registration rate shows a decreasing trend in the eastern region, the central region, and the western region. Table 1 provides the distribution of CDC facilities and the CDC’s government Weibo accounts in mainland China. Of the total 134 accounts, the number of Weibo accounts in the eastern region (n=68, 50.7%) is higher than those in the central region (n=30, 22.4%) and the western region (n=36, 26.9%). There are a total of 158 CDC facilities in the eastern region, and 68 of these have registered government Weibo accounts, with a registration rate of 43.0%. The highest registration rate is in the capital, Beijing (17/17, 100.0%), and the lowest is in Hainan (0/5, 0%). There are 112 CDC facilities in the central region, and 30 of them have registered Weibo accounts, with a registration rate of 26.8%. Henan Province (10/18, 55.6%) has the highest rate, and Heilongjiang Province (0/14, 0%) has the lowest rate. The number of CDC facilities in the western region is 180, of which 36 have registered Weibo accounts, with a registration rate of 20.0%. In the western region, Ningxia (3/6, 50.0%) has the highest registration rate, while Tibet (0/8, 0%) and Qinghai (0/9, 0%) have the lowest rate.

**Table 1:** Distribution of CDC facilities and the CDC’s government Weibo accounts in mainland China.

Province		CDC^a^, n	Government Weibo accounts, n (%)	Registration rate for each location (%)
Total		450	134 (100)	29.8
**Eastern region**		158	68 (50.7)	43.0
	Beijing	17	17 (12.7)	100.0
	Tianjin	17	7 (5.2)	41.2
	Hebei	12	3 (2.2)	25.0
	Liaoning	15	11 (8.2)	73.3
	Shanghai	17	5 (3.7)	29.4
	Jiangsu	14	6 (4.5)	42.9
	Zhejiang	12	8 (6.0)	66.7
	Shandong	17	6 (4.5)	35.3
	Guangdong	22	4 (3.0)	18.2
	Fujian	10	1 (0.7)	10.0
	Hainan	5	0 (0)	0
**Central region**		112	30 (22.4)	26.8
	Hubei	14	4 (3.0)	28.6
	Hunan	15	5 (3.7)	33.3
	Henan	18	10 (7.6)	55.6
	Anhui	17	5 (3.7)	29.4
	Jiangxi	12	1 (0.7)	8.3
	Shanxi	12	4 (3.0)	33.3
	Jilin	10	1 (0.7)	10.0
	Heilongjiang	14	0 (0)	0
**Western region**		180	36 (26.9)	20.0
	Guangxi	15	2 (1.5)	13.3
	Chongqing	39	3 (2.2)	7.7
	Sichuan	22	8 (6.0)	36.4
	Guizhou	10	2 (1.5)	20.0
	Inner Mongolia	13	5 (3.8)	38.5
	Yunnan	17	3 (2.2)	17.6
	Tibet	8	0 (0)	0
	Shaanxi	11	2 (1.5)	18.2
	Gansu	15	5 (3.8)	33.3
	Qinghai	9	0 (0)	0
	Ningxia	6	3 (2.2)	50.0
	Xinjiang	15	3 (2.2)	20.0

^a^CDC: Center for Disease Control and Prevention.

#### Time Distribution of the CDC’s Government Weibo Account Adoption

**Figure 3:**
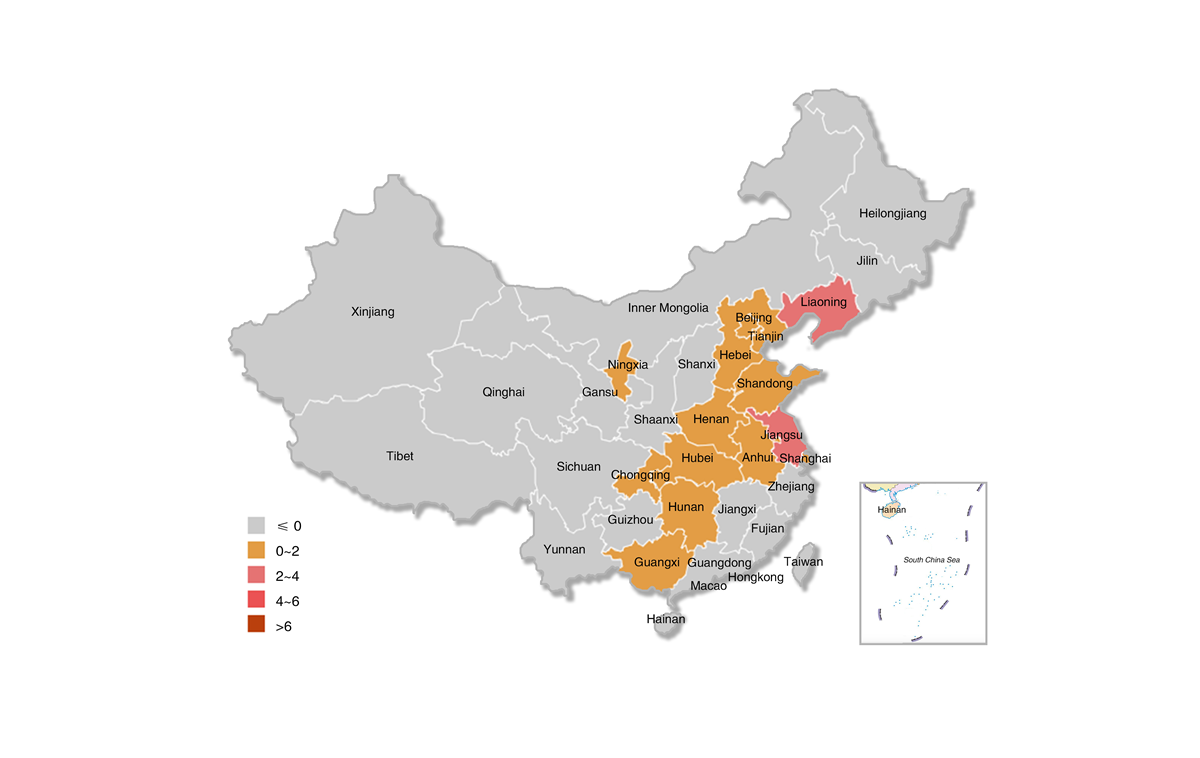
Diffusion of government Weibo accounts (2009-2011).

**Figure 4:**
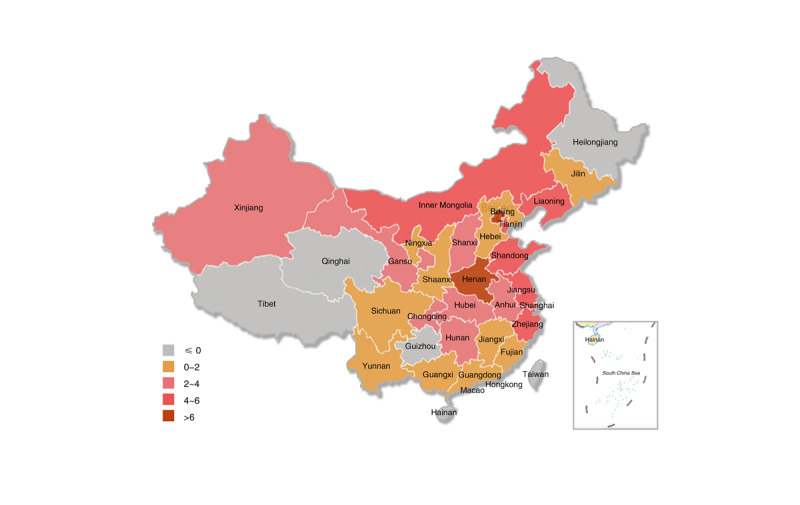
Diffusion of government Weibo accounts (2012-2014).

**Figure 5:**
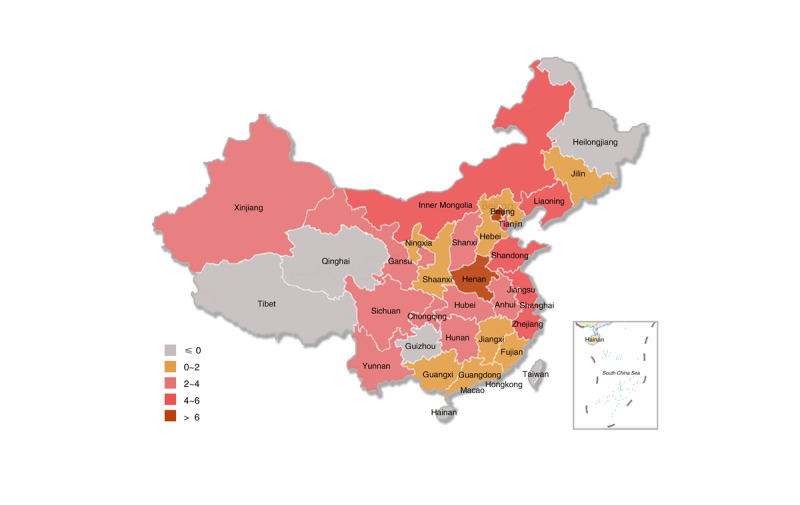
Diffusion of government Weibo accounts (2015-2017).

**Figure 6:**
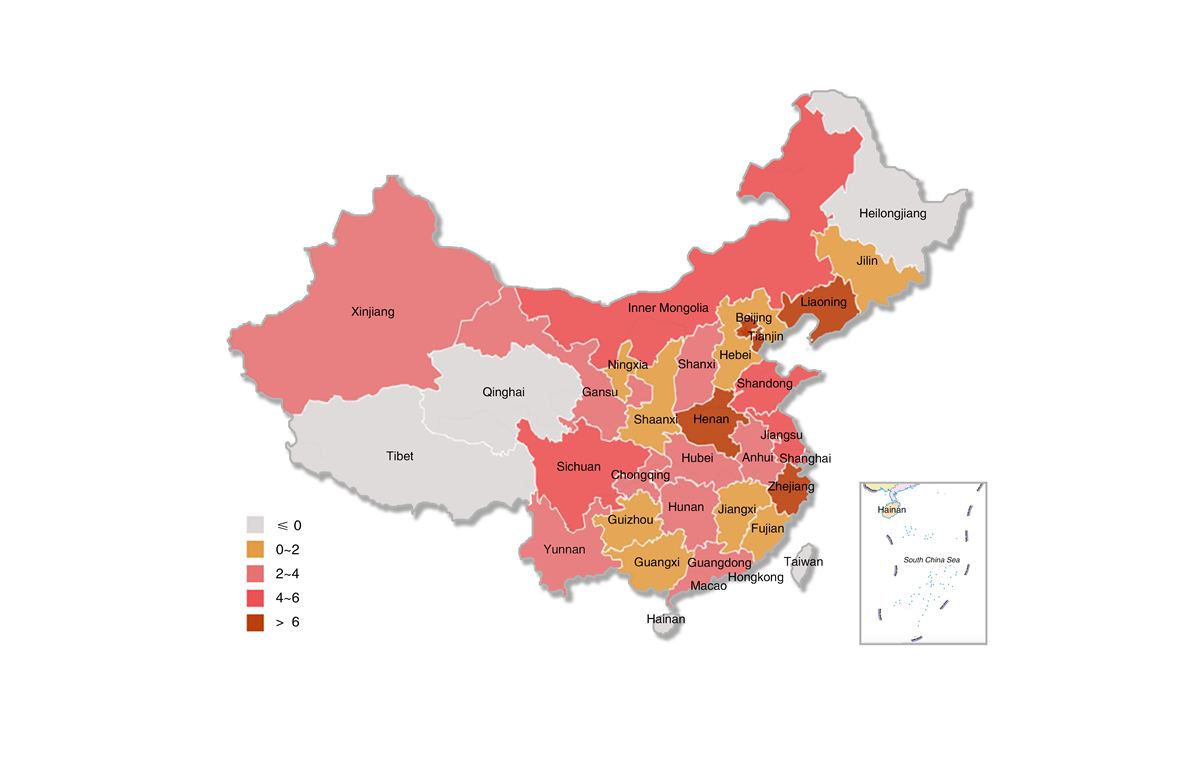
Diffusion of government Weibo accounts (2018-2019).

The CDC’s government Weibo account adoption has a trend of increasing year by year, but there are still some provinces that have not registered government Weibo accounts. The first CDC facility to register a Weibo account in mainland China was the CDC in Lianyungang, Jiangsu Province, which registered on January 17, 2011. Since then, the registration rate of government Weibo accounts has increased year by year. In 2011, of the total 450 CDC facilities, 22 CDC facilities in 14 administrative regions registered government Weibo accounts, with a registration rate of 4.9% (Figure 3). From 2012 to 2014, there were 80 additional CDC facilities with registered government Weibo accounts, increasing the registration rate to 22.7% (102/450), and overall, the accounts were distributed in 26 provincial administrative regions (Figure 4). From 2015 to 2017, there were 24 additional CDC facilities with registered government Weibo accounts, with a registration rate of 28.0% (126/450), and overall, the accounts were distributed in 26 provincial administrative regions (Figure 5). From 2018 to March 2019, there were 8 new CDC facilities with registered government Weibo accounts, increasing the registration rate to 29.8% (134/450), and overall, the accounts were distributed in 29 provincial administrative regions (Figure 6). Currently, the 3 provincial administrative regions of Heilongjiang, Qinghai, and Hainan have not registered government Weibo accounts.

#### Hierarchical Distribution of the CDC’s Government Weibo Adoption

The central-level CDC registered a government Weibo account later than the provincial-level CDC, and the provincial-level CDC registered later than the prefecture-level CDC, showing a “bottom-up” policy learning process. A total of 450 CDC facilities at the provincial and prefectural levels in China have registered Weibo accounts, of which the earliest one is the CDC in Lianyungang, Jiangsu Province, which is at the prefectural level. The registration time was January 17, 2011. On March 31, 2011, the first provincial CDC (Hunan CDC) registered a government Weibo account, which was later than the account registration time of the prefecture-level administrative district. The registration time of the official Weibo, “Science Popularization of Disease Control and Prevention,” of the Chinese Centers for Disease Control and Prevention (as a central institution) was August 23, 2019, which was more than 9 years later than the prefecture-level CDC and provincial administrative regions where Weibo accounts were first registered. In addition, during this period, more than 100 CDC facilities at the provincial and prefectural levels in mainland China registered government Weibo accounts.

### Operation of the CDC’s Government Weibo Accounts

#### The CDC’s Government Weibo Accounts Operating Certification

The Blue V certification is how Sina Weibo authenticates government, media, institutional, and other official accounts, as shown in the bottom right of the Weibo profile photo in Figure 1. This logo shows that Sina Weibo has verified that an account is an organization’s official account, and the main body of the account is more authoritative and authentic, which can help the public to accurately identify official accounts. Of the 134 CDC facilities that have registered Sina Weibo accounts, 88.1% (n=118) of the accounts have been certified with Blue Vs, and the average number of Weibo followers with V-certified accounts is 18,753. There are 16 CDC accounts without certification, and followers do not pay attention to the non–V-certified accounts. One exception is an account that has 12,514 followers; the other Weibo accounts have less than 500 followers.

#### Dropout in the Use of the CDC’s Government Weibo Accounts

The 134 CDC Weibo accounts have different degrees of use and dropout (Table 2). A few of them are “zombie microblogs,” that is, 3.7% (n=5) of the accounts have not posted any content since registering their Weibo account. Some of the accounts are inactive. Only 37.3% (n=50) of the accounts had posted tweets in the past 30 days, 7.5% (n=10) of the accounts had posted tweets in the last 31-90 days, 16.4% (n=22) of the accounts had posted tweets in the last 91-365 days, and 35.1% (n=47) of the accounts had not posted tweets in more than 1 year. Among the latter, most accounts are in the eastern region, accounting for 15.7% (n=21), which is higher than those from the western region at 11.2% (n=15) and those from the central region at 8.2% (n=11). It can be seen that, although the registration rate in the eastern region is relatively high, the dropout rate of more than 1 year is also relatively high.

**Table 2:** The update time of the most recent Weibo tweets from the Center for Disease Control and Prevention’s government accounts (n=134).

Last update time (days)	Eastern region, n (%)	Central region, n (%)	Western region, n (%)	Total, n (%)
≤30	27 (20.1)	12 (9.0)	11 (8.2)	50 (37.3)
31-90	6 (4.5)	1 (0.7)	3 (2.2)	10 (7.5)
91-365	13 (9.7)	5 (3.7)	4 (3.0)	22 (16.4)
>365	21 (15.7)	11 (8.2)	15 (11.2)	47 (35.1)
No content	1 (0.7)	1 (0.7)	3 (2.2)	5 (3.7)

#### Followers of the CDC’s Government Weibo Accounts Were Polarized

The total number of followers on the CDC’s government Weibo accounts was 3,588,544, the average was 26,780 (SD 165,506), and the median was 496. Among the accounts, the Changsha CDC had the largest number of followers, with a total of 1,357,440 followers. The one with the least number of followers was the Zhangye CDC, with a total of 2 followers. The total number of followers for 7 of the CDC’s government Weibo accounts was less than ten; 17 of the CDC’s government Weibo accounts had more than 10,000 followers; and 3 of the CDC’s government Weibo accounts had more than 100,000 followers. These 3 accounts were the Hebei CDC (n=151,289), the Hunan CDC (n=1,331,173), and the Changsha CDC (n=1,357,440).

### Interaction of the CDC’s Government Weibo Accounts

#### Reply Rate to Comments on the CDC’s Government Weibo Accounts

Among the 1215 tweets selected for content analysis in this study, only 12 of the public comments received replies from the government, accounting for less than 1.0%. Statistical analysis of the reply rate and the reply time of the 12 replies found that 50.0% (n=6) of the replies to the comments on Weibo were in the form of “reply only once” and that the remaining 50.0% (n=6) were in the form of “interactive reply.” The response time of 66.7% (n=8) of the Weibo comments was between 0 hours and 1 hour, 8.3% (n=1) of the response times to Weibo comments were between 1 hour and 8 hours, and 25.0% (n=3) of the response times to Weibo comments were more than 12 hours.

#### Influence of the Topic Type on the Interaction

Among the 1215 tweets across all topics, popularizing health knowledge had the most, reaching 606 (49.9%) tweets (Table 3). Disease control information and popularization of disease knowledge both accounted for more than 10.0%. The number of tweets about policy interpretation, emergency response, and Weibo help and citizen consultation had the least, with the number of posts accounting for 0.6% (n=7), 1.2% (n=15), and 1.2% (n=14), respectively. In terms of the number of Weibo retweets, comments, and likes, emergency information posts ranked first, with each post being retweeted 4.1 times, commented on 2.9 times, and liked 4.0 times on average. Policy interpretation received the least number of comments, all of which were 0. This shows that communication effect is better here than for other topic types in dealing with public health emergencies, and it has become the platform for interaction between the government and the public.

**Table 3:** Descriptive statistical analysis results based on the topic type of tweets.

Topic type	Disease control information	Emergency information	Popularization of health knowledge	Popularization of disease knowledge	Radiation hygiene/school hygiene	Government affairs trends	Policy interpretation	Weibo help/citizen consultation	Other
Tweets (n=1215), n (%)	187 (15.4)	15 (1.2)	606 (49.9)	137 (11.3)	24 (2.0)	95 (7.8)	7 (0.6)	14 (1.2)	130 (10.7)
Number of retweets, average	0.4	4.1	0.6	0.7	1.3	0.2	0	1.2	0.3
Number of comments, average	1.0	2.9	0.4	0.3	0.3	0.4	0	1.6	0.3
Number of likes, average	0.7	4.0	0.3	0.5	0.9	0.6	0.9	0.4	0.6

#### Influence of the Content Form on the Interactive Effect

Among the 1215 tweets, the number of posts with “only original text” was 222 (18.3%). The average numbers of retweets, comments, and likes with “only original text” posts were 0.3, 0.3, and 0.3, respectively. On the other hand, there were 993 (81.7%) posts with “pictures/videos/hyperlinks.” The average numbers of retweets, comments, and likes with “pictures/videos/hyperlinks” posts were 0.6, 0.6, and 0.5, respectively. It can be seen that the average numbers of retweets, comments, and likes of microblogs with “pictures/videos/hyperlinks” were higher than those of microblogs with original text, and the interactive effect was better.

#### Influence of the Original Post on the Interactive Effect

Among the 1215 tweets, the number of “original posts” was 703 (57.9%), and the average numbers of retweets, comments, and likes for “original posts” were 0.6, 0.6, and 0.7, respectively. Additionally, the average number of “retweet posts” was 512 (42.1%), and the average numbers of retweets, comments, and likes for “retweeted posts” were 0.5, 0.4, and 0.2, respectively. It can be seen that the average number of retweets, comments, and likes for original posts were higher than those of retweeted posts, indicating that the original content was more in line with the public’s preference.

### Performances of the CDC’s Government Weibo Accounts During COVID-19

#### Activity Rate of the CDC’s Government Weibo Accounts

Of the 134 Weibo accounts, 15.7% (n=21) had a high level of activity, posting more than 5 tweets per day, and 11.2% (n=15) were moderately active, with 1-2 tweets per day. In addition, 23.1% (n=31) posted 1-10 tweets per month. These three categories add up to exactly 50%. However, 46.3% (n=62) of the CDC’s government Weibo accounts have not been updated for more than 1 year, an increase of 11.2% (n=15) over the number of accounts before the epidemic.

#### Main Content of the CDC’s Government Weibo Accounts: COVID-19

Compared with the pre-epidemic statistics, there were approximately 60,000 new tweets and 1.4 million new followers on the 134 CDC accounts. Of the tweets, 90% were about public health emergencies related to COVID-19. These tweets can be divided into four categories. The first is updating the daily epidemic situation including new confirmed cases, new deaths, new suspected cases, new asymptomatic infected people, cumulative cured and discharged cases, life and medical tracking of confirmed cases, etc. The second was announcing the CDC’s work such as the details of procurement announcements. The third was educating the public about the epidemic, including the issuance of protection guidelines for specific places such as schools, companies, shopping malls, and subways; protection guidelines for specific groups such as pregnant women, couriers, taxi drivers, and sanitation workers; nutritional dietary guidelines during the epidemic; and the popularization of disinfectants and protective products. The fourth was publicizing the typical deeds and dedication of the antiepidemic pioneers, especially the CDC.

#### Interaction of the CDC’s Government Weibo Accounts: Decreased Month by Month

At the beginning of the pandemic, the number of posts, comments, and likes was the highest. For example, on January 24, 2020, the tweet “A Letter from Beijing CDC to friends from all over the country who come (return) to Beijing” was released by the Beijing CDC, and the total number of posts, comments, and likes was more than 2000. With the normalization of epidemic prevention and control, the amount of interaction is decreasing. During the epidemic, the questions that the public responded to in the comment area were broadly divided into the following three categories: epidemic prevention and control policies, specific information about new cases, and praising the CDC for its efforts. However, the CDC’s government Weibo accounts still rarely respond to public comments, and only a few of the CDC’s government Weibo accounts provide an office phone number, which is similar to the results of previous studies.

## Discussion

### Principal Findings

An increasing number of public health authorities in China have actively adopted new information platforms and tools for information disclosure and communication, which is the first step in improving communication between the government and the public.

First, the registration rates of the Chinese CDC’s government Weibo accounts in the central and western regions are lower than that in the eastern region. This may be influenced by the government’s motivation and ability to adopt new technologies. According to the “motivation-capability” framework, whether the government adopts new technology or not mainly depends on the motivation and ability of the government; only when that motivation and innovation ability are strong will the new technology be used [23]. Many studies have shown that there is a positive correlation between the level of economic development and the level of government information development [24,25]. Compared with the eastern region, the central and western regions of China are at a geographical disadvantage in terms of economic development, openness, and financial resources [22], so their ability to use government Weibo accounts is also relatively low.

Second, the diffusion process of social media adoption among China’s public health authorities presents two characteristics: one is preferential diffusion among neighboring provinces, that is, horizontal learning and imitation, and the second is the vertical diffusion of local policies from the bottom to the top. Previous studies have shown that due to the influence of the “neighborhood effect,” it is easier for a government to follow the examples of the neighboring leading regions’ governments [26] and learn from the similar experiences of neighboring governments, which helps to improve the success rate of innovation and effectively avoid risks [27]. Previous studies have also confirmed the positive role of the policy learning process [27]. The central government also takes the policy innovation of the local government as the source of policy learning, and once the local policy is successful, it will revise the corresponding policy in time [28].

Third, the social media operations of Chinese public health authorities are still in a passive state. Although nearly 90% of the accounts have official authentication, which can help the public to quickly identify official accounts, and some accounts have a strong ability to reach followers, the overall activity of the accounts was low. Previous studies have shown that the more government is involved in social media operations, the higher the public’s expectation of government interaction [4]. A negative operational status is likely to dampen the public’s enthusiasm for online participation and may not even live up to the public’s relationship expectations [29]. Only by continuously and actively operating social media can we better maintain a normal relationship between public health authorities and the public. Therefore, once the government registers a social media account, it must maintain their social media activity and update daily information frequently.

Fourth, the use of social media by Chinese public health authorities is more inclined to be one-way information dissemination such as popularizing health knowledge, while two-way communication with the public is still limited. For China, where scientific literacy is generally low, the popularization of basic health knowledge is important, but what is more important is how the government mobilizes and communicates using social media to encourage the public to participate in dialogue and cooperation. Especially in the case of limited traditional communication channels, the role of social media is more prominent. Previous studies have indicated that the new dimension that social media brings to the field of public health is that it can change the nature and speed of the interaction between the public and public health authorities [30]. Therefore, governments should use social media not only as a channel to release public health information and transmit health information to the public promptly but also to have two-way dialogues with the public to increase public participation in all stages. This will allow social media to become the best practice for improving communication between the government and the public.

Fifth, the use of social media by Chinese public health authorities provided an important channel of information disclosure and communication for the public during COVID-19, and it generally performed better than before the epidemic, although it still fell short of the Chinese government and public’s requirements. The State Council of China requires that “public messages on the government Weibo should be carefully reviewed, released and processed” [31], but the CDC’s government Weibo accounts tend to be “one-way” by informing the public of the latest developments of the epidemic, and they fail to respond to public inquiries and the large amount of misinformation during the epidemic in a timely manner. In addition, this study shows that the social media interaction effect during the period from January to June 2020 showed a declining trend. This is also consistent with previous research that the government and the public discussion trends in social media can predict the evolution of an epidemic’s dynamics [15]. As the epidemic becomes normalized, the public interest in the dynamics of the epidemic, control policies, and guidelines for prevention and control is waning [32].

Moreover, whether we can maximize the function of social media for public health authorities also depends on the changes of the administrative system and political culture. In China’s centralized political system, the government is dominant in the relationship between the government and the public, and China’s current top-down decision-making and execution mechanism has many bureaucratic levels, which are not conducive to the effective transmission of information. In addition, the common people usually hold the idea of it being “difficult to deal with the government” and are reluctant to communicate with the government; therefore, there is a large psychological distance between the government and the public [20]. Social media provides an opportunity to improve the interaction between the government and the public. Chinese public health authorities must break the thinking mode of the “official standard,” rethink the boundary between the government and the public, and promote the harmonious development of relations between the government and the public.

### Limitations

There are some limitations in this study. First, the survey samples of this study did not include samples of county-level administrative regions (county-level administration regions are governed by prefecture-level administrative regions). Future research can study samples of the county-level administration regions and expand the research results. Second, this study only evaluates the government Weibo accounts; however, government Wechat accounts, as an emerging government social media platform, also have research value. Third, this study only uses descriptive statistics and content analysis, and did not investigate the psychology and behavior of the audience. Future research can use questionnaires, interviews, and other methods to further explore government Weibo use by the audience.

### Conclusions

This study examines the current situation and interaction of social media use by public health authorities in China, a non-Western democratic country. This study analyzes the CDC’s government Weibo accounts for the provincial- and prefecture-level administrative regions in mainland China, and explores how the public health authorities in China improve communication between the government and the public through social media. The results show that the adoption of government Weibo accounts has an uneven regional geographical distribution, steady diffusion year by year over time, and hierarchical bottom-up diffusion. Regarding the operations of government Weibo accounts, nearly 90.0% of government Weibo accounts have official certification, but there are dropouts in the specific operating process. One-third of the accounts have not provided updates for more than 1 year, and the number of microblog followers is polarized, with a maximum and minimum difference of 1 million. Regarding the interaction of government Weibo accounts, although the government Weibo accounts have changed the original layer-by-layer communication mode making communication between the government and the public more convenient, the Chinese government currently is more inclined to release one-way information, and the interaction with the public is limited. The response rate to comments was less than 1%. In terms of the influencing factors, emergency information, multimedia content, and original content are more helpful to promote communication between the government and the public. In the event of a public health emergency such as COVID-19, these accounts can function by updating epidemic information and protection information for the public, although there is still a gap in the two-way interaction. In general, government Weibo use is the first step in improving communication between the government and the public, but the effect is limited and needs to be improved.

## Abbreviations

CDC: Center for Disease Control and Prevention
